# Can Incobotulinumtoxin-A Treatment Improve Quality of Life Better Than Conventional Therapy in Spastic Muscle Post-Stroke Patients? Results from a Pilot Study from a Single Center

**DOI:** 10.3390/brainsci11070934

**Published:** 2021-07-15

**Authors:** Adina Turcu-Stiolica, Mihaela-Simona Subtirelu, Ana-Maria Bumbea

**Affiliations:** 1 Department of Pharmacoeconomics, University of Medicine and Pharmacy of Craiova, 200349 Craiova, Romania; mihaela.subtirelu@yahoo.com; 2 Department of Physical and Rehabilitation Medicine, Faculty of Medicine, University of Medicine and Pharmacy of Craiova, 200349 Craiova, Romania; anamariabumbea@yahoo.com

**Keywords:** incobotulinumtoxin-A, post-stroke, spasticity

## Abstract

Post-stroke spasticity frequently occurs in patients with stroke, and there is a need for more quality-of-life assessments for different therapies. We evaluated for the first time in Romania the quality of life among patients with post-stroke spasticity, comparing two therapies over a 6-month period: botulinum toxin type A (BOT) with conventional therapy (CON). We also assessed the reduction of spasticity and functionality secondary to the increase in the mobility in upper limbs. This study was based on a prospective, randomized design, including subjects with post-stroke spasticity (N = 34; 34–80 years of age): in the CON arm, patients received therapy against muscle spasticity and physiotherapy, and, in the BOT arm, patients received incobotulinumtoxin-A and additionally conventional treatment, if required. Among 34 treated subjects in the two arms, the quality of life was significantly higher after BOT therapy (*p* < 0.001), represented by improvement in movement (*p* < 0.001), usual activities (*p* = 0.018), and distress (*p* < 0.001). Improvements in muscle tone (Ashworth Scale) over 6 months of treatment period were greater in the BOT arm (100%) than in the CON arm (11.8%). These preliminary results suggested that incobotulinumtoxin-A increased quality of life by improving movement, daily activities, mental health, and muscle tone more effectively than conventional therapy and could form a basis for future comparator studies.

## 1. Introduction

The quality of life of the stroke patient who survives the acute episode is an important goal of modern medicine [1]. Thus, we aimed to draw attention to the impacts on different aspects of daily life, by increasing a patient’s mobility capacity following a stroke via the application of a specific focal therapy to combat spasticity [1,2].

Stroke is a leading cause of morbidity and mortality globally. In underdeveloped countries, the incidence is higher in terms of morbidity and, additionally, mortality, due to the risk factors of arterial hypertension, atrial fibrillation, abandoned anticoagulant treatment, and untreated dyslipidemia [3,4].

Globally, the incidence of stroke is the second leading cause of mortality. Similarly, in Romania, stroke remains the second most disabling cause of mortality and the leading cause following the onset of dementia [4]. The disability imposed by stroke is due to both motor deficits and exacerbated spasticity, which is specific to pyramid syndrome. Residual spasticity poststroke is the main disabling cause, and limits the realization of active mobility [4,5].

There are several types of spasticity therapies, such as oral drugs, physiotherapy, and focal therapies with botulinum toxin. In our research, we applied botulinum toxin therapy as a focal therapy via local injection into the spastic muscles of the upper limb [6]. It should also be noted that botulinum toxin therapy does not present risk in post-COVID-19 patients, and is a therapy that can be applied without restrictions. An opinion article discussed the treatment of spasticity during the COVID-19 pandemic and noted that botulinum toxin therapy has not been recommended for discontinuation or interruption and cannot be postponed. This therapy should be performed at cyclic intervals. In COVID-19 patients or during the period of limited access to treatment in hospitals, certain clinical criteria may be considered. The authors propose to consider the factors of time over 3 months since the last administration; the clinical evaluation; the increased degree of spasticity with resonance over time if the administration time is exceeded; the pain; and the impossibility of using orthoses. The therapy administered must comply with the rules in force during the pandemic for safety using special personal protective equipment [7]. Furthermore, Mahesh Kandasamy evaluates the safety profile of botulinum toxin as a therapy in reducing respiratory syndrome in patients with COVID-19 with failure respiratory syndrome [8].

A cost-effectiveness analysis of treating post-stroke patients with upper limb spasticity was undertaken in Romania, highlighting the Quality-Adjusted Life Year (QALY) gained for incobotulinumtoxin-A compared with conventional therapy. In addition, using a mapping algorithm, the results were compared with the values for the quality of life from other studies [9]. The direct and indirect costs of stroke have been assessed in Romania, and found to be dominated by direct costs [10,11], with important cost differences between post-stroke therapies [12]. Economic evidence is not sufficient to support policy decisions to fund new interventions and a need exists for of quality of life (QoL) evaluation. This study aimed to investigate for, the first time in Romania, the differences in quality of life between post-stroke patients treated with botulinum toxin and conventional therapy. We also aimed to assess the reduction of spasticity and functionality secondary to the increase in the mobility in the upper limb.

## 2. Materials and Methods

### 2.1. Ethical Issues

This prospective, randomized study (randomization ratio 1:1) was conducted according to the Helsinki Declaration regarding informed consent and confidentiality. All patients gave signed informed consent via a form at the hospital upon admission. The study was approved by the University of Medicine and Pharmacy of Craiova Ethics Commission (no. 74/21.05.2020).

### 2.2. Participants

This single center study involved inpatients and outpatients with post-stroke spasticity with a minimum of 3 months after stroke onset, at which point the evaluation and stratification into study arms began. The reporting of study findings follows the STrengthening the Reporting of OBservational studies in Epidemiology (STROBE) criteria [13]. The patients were enrolled from the Neurology Hospital of Craiova, Romania, during the period from May 2020 to February 2021.

Inclusion criteria were: age ≥ 18; ischemic or hemorrhagic stroke (as documented radiologically by a computerized tomography scan or magnetic resonance imaging; subarachnoid hemorrhage excluded); time since stroke onset ≥3 months (the limit of 3 months was chosen because spasticity occurs at least 6 weeks after the onset of stroke); Ashworth scale ≥ 2; no previous focal treatment of post-stroke spasticity with botulinum toxin; no other antispastic medications (including muscle relaxants).

The limitation of the number of patients used in this study was imposed by the conditions of the COVID-19 pandemic, which restricted the admission and addressability in the neurological recovery service. The necessary measures were taken to maintain the spacing and security conditions with safe medical services. Patients completed a specific triage questionnaire to be admitted to the hospital and performed a specific treatment. No patient had SARSCoV-2 infection.

Exclusion criteria were: neurologically, cardiological, or respiratory unstable patients were not admitted, respiratory pathology was excluded because the risk of respiratory depression may be amplified by the administration of botulinum toxin; other orthopedic conditions involving the affected limbs. Patients who had contraindications to botulinum toxin injection were excluded, such as patients receiving anticoagulant therapy, patients with myasthenia, or patients with skin disorders at the injection site.

### 2.3. Methodology

The study compared two patient groups: one arm included patients receiving specific stretching exercises and incobotulinumtoxin-A (BOT) injections without any conventional (CON) therapy for spastic muscle, and another arm included patients receiving CON therapy for muscle spasticity and physiotherapy. Data from both groups were collected two times: at baseline, before antispastic treatment (T0), and after 6 months (T1). Patients were evaluated clinically, cognitively, and functionally. At baseline, data on age, gender, stroke subtype, marital status, employment status, environment, and education were obtained.

#### 2.3.1. Measures

The spasticity Modified Ashworth Scale (MAS), Tardieu, and Frenchay scale were used for functional assessment. The MAS score assumes integer values between 1 and 4, and grades the resistance of a relaxed limb to rapid passive stretching (1 = slight increase in muscle tone manifested by a catch and release or by minimal resistance at the end of the range of motion; 1+ = slight increase in muscle tone manifested by a catch, followed by minimal resistance throughout the remainder, less than half, of the range of motion; 2 = more marked increase in muscle tone through most of the range of motion; 3 = considerable increase in muscle tone, passive movement difficulty; 4 = affected part is rigid) [14].

The Tardieu scale is used to quantify spasticity in the neurological patient in which the muscular reaction to a movement with different speed is evaluated: fast or slow, with the observation of the muscular reaction and the angle of muscle reaction. The gradation is from 0 to 4: 0 no resistance to passive movement, 1—slight resistance on the passive movement, 2—catch reaction at a specific angle with the interruption of the passive movement and the relaxation, 3—the appearance of fatigable clonus less than 10 s, 4—the appearance of the clonus for more than 10 s at the same passive movement at a specific angle [15].

The Frenchay Scale or Frenchay Activity Index (FRENC) is a method of measuring daily instrumental activities, IADL. The variant applied in our study was the modified form of the Frenchay Scale, i.e., the Frenchay Arm Test which includes 10 activities and 6 mono-manuals for the upper limb: objects placed in a semicircle in front of the patient, from larger objects to small objects and finesse activities [16].

Activities of daily living (ADL) include maintaining hand hygiene by hand washing and wiping, changing clothes, standing up, and walking.

The Barthel Index is an assessment of disability and uses 10 activities for both the upper limb and the lower limb to highlight the degree of disability. The scoring is different for each activity and has a maximum of 100 points [17].

Muscle strength (MS) was evaluated using muscle testing with a score from 0 to 5, where 0 represents the absence of muscle tone, 1—the presence of tone but without the ability to perform movement, 3—movement is possible horizontally, 4—movement is possible in an antigravitational plane, without resistance, and 5 represents the capacity of antigravitational movement against a resistance and corresponds to the normal muscle [18].

The Mini Mental State Examination (MMSE) is a test that includes 30 questions to assess cognitive impairment. It must be undertaken within a maximum of 10 min, and the maximum score is 30 points. A score of over 25 and up to 30 is considered to be a normal score [19].

#### 2.3.2. 15D-Instrument

The health-related quality of life (HRQoL) was assessed using the Romanian version of the general instrument 15D [20]. This instrument captures multidimensional effects from the perspective of the patient, which is clinically important information for the rehabilitation services. The instrument includes 15 items, which are summed in a final score valued from 0 to 1, where 0 equals death and 1 means perfect health. The items include mental and physical burdened patients on mobility, vision, hearing, breathing, sleeping, eating, speech, excretion, usual activities, mental function, discomfort and symptoms, depression, distress, vitality, and sexual activity. Each domain is evaluated using a 5-point Likert scale in which higher scores indicate better function. Subjects completed the 15D-instrument at the baseline visit and at 6 months post-injection/conventional treatment.

### 2.4. Treatment

For both arms, anti-spasticity therapy was applied by physical and medication therapy. The difference between the groups consisted of the type of medication therapy applied.

The selected groups were numerically equal and were divided into the BOT group, which received physiotherapy and applied focal spasticity therapy using botulinum toxin type A: incobotulinumtoxin-A (INCO, Xeomin^®^), and the CON group received physiotherapy and oral drug treatment of spasticity: baclofenum (started from 10 mg up to 60 mg daily).

In this study, we considered conventional therapy—a different type of therapy than that consisting of the administration of botulinum toxin.

The final evaluation was chosen to be at 6 months because during this interval the patients of the BOT group followed two cures of INCO. This was a special regimen of administration and was able to be administered for at least 3 months. Patients in the CON group received physical therapy and the specific physiotherapy program for spasmodic muscles with the readjustment of the physical program at 3 months to respect the study design.

**Figure 1:**
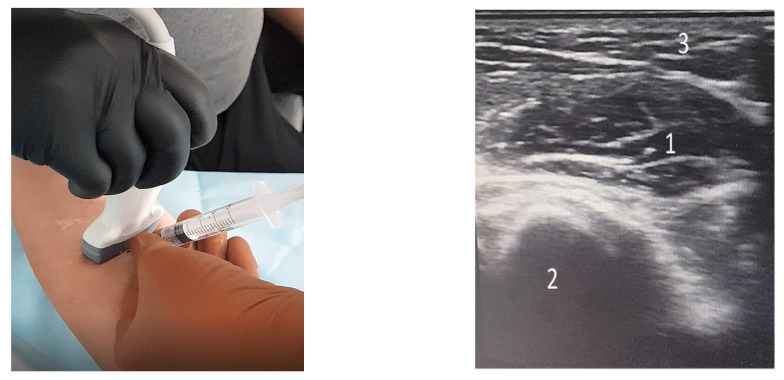
Incobotulinumtoxin A injection of the right upper limb—pronator teres muscle. The ultrasound image shows the pronator teres muscle (1), to be injected, and adjacent structure: ulna bone (2) and subcutaneous tissue (3). On the gray scale, the muscle appears more hypo echoic, bordered by hyper echoic lines, representing the muscular fascia. Ulna bone—only the edge that appears intense hyper echoic-white is visualized, the rest of the bone structure not being visible by ultrasound and the shadow cone appears-black-under the bone. The subcutaneous tissue is hypo echoic and located on the surface, above the muscle.

The BOT group received a specific program of stretching exercises for the spastic muscles of the upper limb. Focal spasticity therapy consisted of injecting therapeutic doses of INCO into the target muscles. The injection was performed only on the upper spastic limb, as in Figure 1. The administration of botulinum toxin followed the corresponding dose of 200 U for INCO.

The CON group, which were in the hospitalized system, received a specific classic specific physical kinetic treatment which consisted of electrotherapy to stimulate the paralyzed muscles combined with elements of kinetotherapy and stretching applied to the spastic muscles and antispastic drug treatment of baclofenum. The BOT patients were in the hospitalized system only for administration of INCO and they also received kinetotherapy.

### 2.5. Statistical Analysis

All statistical analysis was performed using the GraphPad Prism 9.1.2 software (GraphPad Software, San Diego, CA, USA). Descriptive statistics were presented as percentages for categorical variables and mean ± standard deviation (SD) for continuous variables. Percentages were calculated using the denominator of the subjects in every treatment group. The two groups were compared using the Chi-square test for categorical variables and Mann-Whitney U test for continuous variables. *p*-values less than 0.05 were considered statistically significant.

## 3. Results

A study sample of 34 stroke patients was included in this study. All 34 patients completely answered the questionnaires. Data on age, gender, marital status, employment status, education level, comorbidities (hypertension, diabetes, ischemic heart disease), and stroke subtype, at baseline, are shown in Table 1. The gender of patients is similar with a mean (SD) age at baseline of 60.9 (SD = 12.9) years, with older patients in CON group (*p* = 0.002). All patients were retired due to the age (range = 34–80) or spasticity.

The two groups had the same distribution for marital status (all the patients were married) and environment status (*p* = 0.49).

### 3.1. Health Characteristics

The type of stroke was mostly ischaemic, without statistically significant differences between the two groups (*p* = 0.688). The patients suffered from simultaneous diseases, most commonly from hypertension (97%) and ischemic heart disease (92%). Table 2 reports the mean, standard deviations, and percentages of all variables for the health status of the patients. Improvements in muscle tone (Ashworth Scale) over 6 months were greater in the BOT arm (100%) than in the CON arm (11.8%).

**Figure 2:**
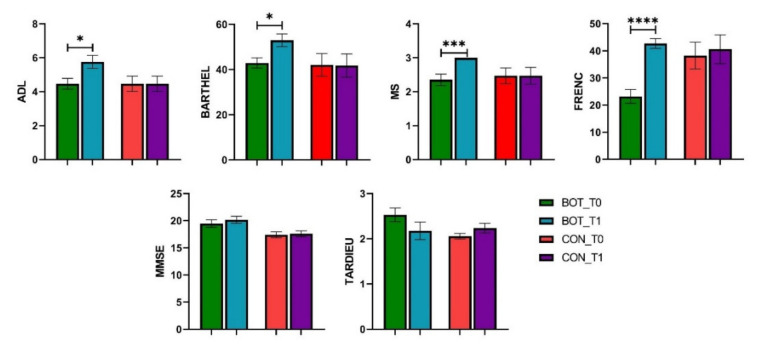
Differences between baseline and study end for BOT and CON groups (mean and SEM). *, p-value < 0.05; **, p-value < 0.001; ***, p-value < 0.0001

Measures changes from baseline to study end are shown in Figure 2. Testing of ADL (*p* = 0.0174), BARTHEL (*p* = 0.0119), MS (*p* = 0.0009), and FRENC (*p* < 0.0001) for the BOT group yielded statistically significant differences between baseline and study end, whereas MMSE (*p* = 0.4031) and TARDIEU (*p* = 0.1754) remained almost the same. In the case of the CON group, no statistically significant differences occurred between baseline and study end.

### 3.2. Baseline and Post-Treatment Changes in 15D Scores

Data quality in the completed questionnaires was good, without missing data. Participants in the BOT group at baseline reported significantly more functional problems in the speech domain, whereas in the CON group, patients experienced sight problems. In the BOT group, we observed a statistical improvement of some dimensions of quality of life: move (*p*-value < 0.001), usual activities (*p*-value = 0.018), and distress (*p*-value < 0.001), with an enhancement of the total 15D score. Table 3 contains the descriptive statistics for each health component and dimensions of the 15D-instrument, for each very measurement time.

**Figure 3:**
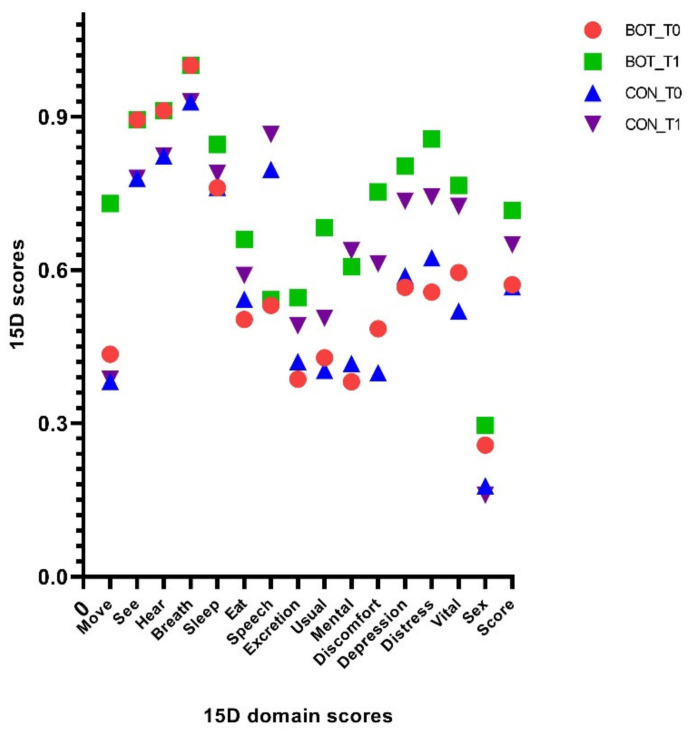
The mean 15D profiles for BOT and CON groups at baseline and study end.

Figure 3 compares the mean values obtained for all 15 dimensions of the QoL instrument: mobility, vision, hearing, breathing, sleeping, eating, speech, excretion, usual activities, mental function, discomfort and symptoms, depression, distress, vitality, and sexual activity. Comparing the two groups, the largest differences were observed in moving, usual activities and distress dimensions. The quality of life at baseline was the same for BOT group patients and CON group patients (*p* = 0.838), but it increased more after the treatment with BOT (0.72 vs. 0.68, *p* < 0.001).

## 4. Discussion

To our knowledge, this prospective study is the first evaluation of quality of life of BOT in comparison with CON, with respect to upper limb spasticity in Romania. The cost-utility analysis of BOT compared with CON in the management of post-stroke spasticity in Romania was undertaken by mapping data for QoL assessment from other published studies [21]. We obtained larger increases in quality of life in the BOT group than those obtained in other studies obtained [22]. Changes in patients receiving BOT were significantly enhanced for ADL, BARTHEL, FM, and FRENC, whereas MMSE and TARDIEU did not show improvement.

The novelty and originality of this study also consist in the assessment of cognitive aspects, and not only functional status, secondary to the reduction of excessive spasticity in the stroke patient. The distress score improved under BOT treatment, better than under COV treatment. Interestingly, using the SF-12 tool for assessing the QoL, another study found that the mental score was improved under both treatments [21]. The paucity of data on QoL, especially mental health, in subjects with chronic spasticity, is a major limitation, and specific tools with various dimensions that must be developed and validated.

In 2014–2015, Barnes and colleagues conducted a survey of the impact of spasticity on daily life on a large cohort of patients with spasticity, who were evaluated via an Internet survey. Their results reported a 72% impact on quality of life with up to 44% loss of independence. In addition, the same percentage was recorded for depression in patients with spasticity. This aspect of the change in the quality of life of the patients with spasticity was also the motivation to evaluate the patients′ quality of life following the application of INCO therapy. Conventional therapy failed to obtain the same score in the assessment of the patient′s quality of life, a finding consistent with that of Barnes [23].

In two studies, ULIS I and ULIS II, conducted over a 10-year period, Turner-Stokes and colleagues showed the importance for the quality of life of managing spasticity of the upper limb in stroke patients. The project, which involved 84 care centers in 22 countries and the participation of 456 patients with spasticity, confirmed the importance in daily life of spasticity management for current activities. The publication of the project data in 2019 drew attention to the long-term necessity of the therapy, and the perseverance of both specialists and patients to obtain and maintain the results. The 10-year evaluation period of the study indicates the concern for the quality of life of the patients with spasticity and the sustained therapy, an aspect that is highly important for improvement of functionality [24,25]. Our study presented the same result in terms of spasticity. In addition, the novelty offered by our study is the greater improvement in the patient′s quality of life following the focal therapy of spasticity compared to the conventional therapy.

Another study that examined the effects of botulinum toxin type A injections over a 12-year period showed that starting therapy as early as possible has long-term beneficial effects. The published data confirm the safety and efficacy of the therapy and spasticity of various etiologies over a long period of time. Moreover, the data imply that treatment with botulinum toxin A should be started earlier after the onset of spasticity to obtain a better response to therapy. This information is important for deciding and choosing when to start treatment, in patients with spasticity [26].

A phase III Japanese study published in 2020, J-PURE, was conducted to evaluate the efficacy and safety of botulinum toxin type A with doses up to 400 U for treatment of upper limb spasticity, and provides current information relating to this type of treatment. The 12-week, double-blind, placebo-controlled study shows that active movement improves in the study group that received treatment with botulinum toxin type A. Furthermore, the scale of disability and clinical evaluation according to the examiner′s overall impression show that INCO reduced increased muscle tone, improved functionality, and was well tolerated in Japanese subjects with upper limb spasticity after stroke [27].

A global study in 2016, in the adult population, showed the frequency of body segments affected by spasticity: 13% damage to the whole body, 44% damage to the upper limbs, and 49% damage to the lower limbs. More than half of the cases were due to stroke (47%) and brain damage. These data confirm the approach for patients with spasticity in the context of our study, which validates the improvement of several aspects of the quality of life by reducing spasticity and continuing therapy cycles [23].

Another phase III study, OLEX, which included three cycles of INCO treatment, given at fixed intervals of 12 weeks, was published in 2019. A quantity of 400 U INCO was administered to the affected upper limb. Efficacy assessments included assessing muscle tone using the Ashworth Scale and the Global Impression of Change Scale assessed by the investigator, subject, and caregiver. The study included 299 patients, of whom 248 reached the end of the 36-week study. Results were obtained by selective evaluations of each muscle injected in the three cycles of treatment, and highlighted both the efficacy and safety of focal spasticity therapy by injecting INCO [28].

A placebo-controlled, double-blind, multicenter study by Kanovsky et al., studied improved muscle tone and upper limb function for a minimum of 20 weeks, with an extension of up to 89 weeks, in a cohort of 145 patients. The results demonstrated continuous and significant sustained improvements in muscle tone and reductions in the degree of disability [29].

However, a review from 2019 followed the functional choices of focal treatment with botulinum toxin on upper limb spasticity using the MEDLINE and Cochrane databases for studies, reviews, and meta-analyses. The presented review focused on spasticity, in addition to the disability induced by stroke-generating spasticity. Although the disability assessment scale and goal achievement scale were followed, the results failed to show greater improvement after the injection of botulinum toxin type A. The authors showed that the relationship between decreased spasticity and improvement in upper limb activity was not established for a number of reasons: due to muscle hypotonia rather than spasticity; patients were unable to benefit from focal treatment with botulin toxin type A; or the assessment methods could not be adapted for all eligible patients. A final aspect noted by the authors was the short-term monitoring period, i.e., a single treatment cycle, which did not allow sufficient time for establishment of rehabilitation programs. The purpose of this review was to highlight the need for upper limb rehabilitation to achieve functional outcomes, and thus enhance the functional effects of botulinum toxin type A injection [30].

The strengths of our research are based on the complex evaluation of the patients, who received adapted rehabilitation therapy and different therapies for spasticity. Each patient received a kinetic protocol consisting of exercises specific to each spastic muscle that required injection of botulinum toxin type A. Good results were obtained in the BOT group, which received, in addition to focal therapy, recovery treatment using serial exercises specific to each muscle. These results demonstrate the need for the association of the two types of treatment, thus ultimately resulting in improvements in the quality of life.

Given the limited number of patients, the results reported here will serve as a valuable starting point for further research in this direction.

## 5. Conclusions

Incobotulinumtoxin-A was found to improve quality of life better than conventional therapy in spastic muscle post-stroke patients, especially in moving, daily activities and distress.

## Figures and Tables

**Table 1:** Demographics of the patients included in this study.

Characteristics	Patient Group				*p*-Value
	BOT (N = 17)		CON (N = 17)		
	Count	Mean (±SD)	Count	Mean (±SD)	
Age, years		59.53 (±8.94)		60.91 (±12.86)	0.002 *
Gender					1.00
Men (n = 21)	9 (52.9%)		8 (47.1%)		
Women (n = 13)	8 (47.1%)		9 (52.9%)		
Married (yes)	17 (100%)		17 (100%)		1.00
Employment status					1.00
Retired	17 (100%)		17 (100%)		
Environmental					0.494
Rural	10 (58.8%)		7 (41.2%)		
Urban	7 (41.2%)		10 (58.8%)		
Education					0.001
Faculty			1 (5.9%)		
Highschool	17 (100%)		7 (41.2%)		
Elementary school			9 (52.9%)		

Abbreviations: BOT, incobotulinumtoxin-A treatment; CON, conventional therapy; SD, standard deviation; Statistically significance represented by asterisk: *, *p*-value < 0.05.

**Table 2:** Health status of the patients.

	Patient Group				
	BOT (N = 17)		CON (N = 17)		
	Count	Mean (±SD)	Count	Mean (±SD)	*p*-Value
Type					0.175
Ischaemic	12 (70.6%)		16 (99.94%)		
Hemorrhagic	5 (29.4%)		1 (0.06%)		
Hypertension (yes)	16 (94.1%)		17 (100%)		0.310
Diabetes (yes)	2 (11.8%)		9 (52.9%)		0.026
Ischemic heart disease (yes)	14 (82.4%)		17 (100%)		0.227
Jacksonism (yes)	4 (23.5%)		3 (11.8%)		1.000
Haemipareshis (right)	10 (58.8%)		5 (29.4%)		0.166
Ashworth_T0					0.001
1	0		2 (11.8%)		
2	1 (5.9%)		11 (64.7%)		
3	10 (58.8%)		3 (17.6%)		
3/4	6 (35.3%)		1 (5.9%)		
Ashworth_T1					0.198
1	4 (23.5%)		2 (11.8%)		
2	8 (47.1%)		9 (52.9%)		
2/3	3 (17.7%)		0		
3	2 (11.8%)		5 (29.4%)		
4	0		1 (5.9%)		
ADL_T0		4.47 (±1.33)		4.47 (±1.58)	0.413
ADL_T1		5.76 (±1.56)		5.12 (±1.81)	0.099
Barthel_T0		42.94 (±9.36)		42.50 (±15.82)	0.786
Barthel_T1		52.94 (±11.6)		47.35 (±17.81)	0.092
MS_T0		2.35 (±0.70)		2.41 (±0.82)	0.563
MS_T1		3.00 (±0.00)		2.74 (±0.75)	0.150
MMSE_T0		19.47 (±2.85)		18.44 (±2.72)	0.041 *
MMSE_T1		20.18 (±2.68)		18.88 (±2.77)	0.008 *
Tardieu_T0		2.53 (±0.62)		2.29 (±0.52)	0.038 *
Tardieu_T1		2.18 (±0.81)		2.21 (±0.64)	0.76
FRENC_T0		23.18 (±10.45)		30.71 (±17.69)	0.031 *
FRENC_T1		42.71 (±7.53)		41.65 (±16.18)	0.540

Abbreviations: ADL, Activities of daily living; MS, Muscle strength; FRENC, Frenchay Scale; MMSE, Mini Mental State Examination. Statistically significant represented by an asterisk: *, *p*-value < 0.05.

**Table 3:** Baseline and post-treatment changes in QoL for post-stroke patients.

Dimensions	BOT (Mean ± SD)			CON (Mean ± SD)			*p*-Value for T0	*p*-Value for Difference between Scores
	T0	T1	Difference between Scores	T0	T1	Difference between Scores		
Move	0.44(±0.12)	0.69(±0.12)	0.09(±0.13)	0.41(±0.12)	0.54(±0.21)	0.13(±0.16)	0.306	<0.001 *
See	0.89(±0.15)	0.89(±0.15)	0	0.84(±0.13)	0.84(±0.13)	0	0.012 *	1.000
Hear	0.91(±0.12)	0.91(±0.12)	0	0.87(±0.13)	0.87(±0.13)	0	0.079	1.000
Breath	1	1	0	0.96(±0.09)	0.96(±0.09)	0	0.245	1.000
Sleep	0.76(±0.09)	0.85(±0.12)	0.06(±0.11)	0.76(±0.06)	0.82(±0.12)	0.06(±0.12)	1.000	0.274
Eat	0.50(±0.16)	0.66(±0.24)	0.12(±0.17)	0.52(±0.14)	0.62(±0.20)	0.10(±0.15)	0.586	0.053
Speech	0.53(±0.29)	0.54(±0.28)	0.02(±0.07)	0.66(±0.29)	0.70(±0.29)	0.04(±0.09)	0.009 *	0.357
Excretion	0.39(±0.19)	0.55(±0.23)	0.11(±0.16)	0.40(±0.17)	0.52(±0.22)	0.11(±0.14)	0.496	0.140
Usual Activities	0.43(±0.19)	0.68(±0.15)	0.10(±0.14)	0.42(±0.17)	0.59(±0.19)	0.18(±0.16)	0.865	0.018 *
Mental Function	0.38(±0.15)	0.61(±0.25)	0.19(±0.15)	0.39(±0.14)	0.62(±0.21)	0.22(±0.11)	0.496	0.838
Discomfort	0.48(±0.22)	0.75(±0.24)	0.25(±0.12)	0.44(±0.19)	0.68(±0.21)	0.24(±0.16)	0.357	0.540
Depression	0.57(±0.17)	0.75(±0.20)	0.16(±0.12)	0.58(±0.14)	0.74(±0.16)	0.16(±0.12)	0.760	0.760
Distress	0.56(±0.18)	0.86(±0.17)	0.11(±0.14)	0.59(±0.16)	0.79(±0.15)	0.21(±0.14)	0.357	<0.001 *
Vitality	0.59(±0.17)	0.77(±0.21)	0.14(±0.15)	0.56(±0.15)	0.74(±0.17)	0.19(±0.12)	0.205	0.122
Sexual Activity	0.26(±0.13)	0.29(±0.18)	0.00(±0.15)	0.22(±0.12)	0.23(±0.15)	0.01(±0.09)	0.085	0.193
Total 15D score	0.57(±0.12)	0.72(±0.14)	0.13(±0.05)	0.57(±0.09)	0.68(±0.12)	0.11(±0.05)	0.838	<0.001 *

Statistically significance represented by asterisk: *; the significance level is 0.05.

## Data Availability

The datasets used and/or analyzed during the current study are available from the corresponding author on reasonable request.
